# Case report: A rare case of a long-term survivor of glioblastoma who underwent two courses of hypofractionated radiotherapy as part of her care

**DOI:** 10.3389/fonc.2025.1501466

**Published:** 2025-02-04

**Authors:** Midhad Mrvoljak, Shubhendu Mishra, Liam Chen, Elizabeth Neil, Eric Ehler, Stephanie Terezakis, Lindsey Sloan

**Affiliations:** ^1^ College of Osteopathic Medicine, Des Moines University, Des Moines, IA, United States; ^2^ Department of Radiation Oncology, University of Minnesota Medical School, Minneapolis, MN, United States; ^3^ Masonic Cancer Center, University of Minnesota, Minneapolis, MN, United States; ^4^ Department of Pathology, University of Minnesota Medical School, Minneapolis, MN, United States; ^5^ Department of Neurology, University of Minnesota Medical School, Minneapolis, MN, United States

**Keywords:** glioblastoma, long-term survivor, hypofractionated radiotherapy, case report, reirradiation

## Abstract

Glioblastoma (GB) is a primary brain tumor that is lethal and challenging to treat. The 3-year overall survival (OS) of patients with this diagnosis has stayed the same since 2005. The patient is a 75-year-old woman who presented with progressive aphasia and was diagnosed with GB (WHO grade 4, IDH1/IDH2 wild type, ATRX intact, p53 and PTEN mutant, BRAF non-mutated, O^6^-methylguanine-DNA methyltransferase promoter methylated) and who underwent surgical resection, hypofractionated radiotherapy (HFRT) using intensity-modulated radiotherapy (IMRT) (4,005 cGy in 15 fractions) alone, and adjuvant temozolomide (TMZ). She was progression-free for approximately 20 months. Although planned, concurrent TMZ was not used during the complete first course of HFRT due to the patient’s performance status. After recurrence, another HFRT (35 Gy in 10 fractions) was employed. She was progression-free on imaging for 8 months until a recent follow-up scan showed potential progression *versus* radiation-related change. At the time of this case report, her care is still ongoing. This represents a rare case of a long-term survivor of GB who has received two courses of HFRT, a treatment option that is usually used in those with predicted shorter survival times.

## Introduction

Glioblastoma (GB) is a primary brain tumor arising from supporting glial cells within the central nervous system and affects about three per 100,000 people per year, with a 3-year survival rate of about 10% (1, 2). For patients over the age of 65 years old, survival at 3 years is even lower, about 5% (1). There is no standard definition of a long-term survivor of GB, but Briceno et al. suggests that a duration of 3 years from diagnosis may be one way to identify this exceptional population (3).

The mainstay treatment for GB is surgery with adjuvant radiotherapy (RT) of 60 Gy in 30 fractions and concurrent temozolomide (TMZ) that produces an overall median survival of 14.6 months (2). Unfortunately, although they have been shown to extend life through the highest level of clinical evidence, each modality is associated with potential side effects. The side effects of surgical resection include bleeding, cognitive impairment and/or functional impairment, seizures, and infection (4). Chemotherapy, with standard of care of TMZ, may result in hematological changes such as lymphopenia, thrombocytopenia, and/or neutropenia (2). The common acute side effects of RT are fatigue, nausea, hair loss, and cerebral edema (5, 6). Serious long-term side effects of RT include cognitive decline and radionecrosis (5, 6). Overall, these treatments are reasonably well tolerated, with only 16% of patients experiencing grade 3 or 4 hematologic side effects from chemoradiotherapy (2). There were only two deaths from cerebral hemorrhage out of 284 patients who received the Stupp protocol, likely attributable to RT with TMZ (2).

Ultimately, the low survival rate in patients with a diagnosis of GB reflects the lack of a cure as GBs inevitably recur; therefore, factors such as duration of treatment must be balanced with quality of life (QOL). Positive prognostic factors of survival in patients with GB have been reported. The most impactful of these include a favorable location of the tumor, high extent of resection, better performance status, younger age, and O^6^-methylguanine-DNA methyltransferase (MGMT) promoter methylation. It is worth noting that MGMT promoter methylation is important even in older individuals only (7, 8). These factors play a role in decision making with patients afflicted by GB. A randomized phase 3 trial showed that patients greater than 65 years old with MGMT promoter methylation had a longer event-free survival when treated with TMZ (9).

With very few patients experiencing long-term local control after initial therapy, additional lines of anti-cancer treatment may be considered at the time of GB recurrence, which is usually within the prior RT field (10). At the time of pathological or imaging a suspected recurrent GB, the patients are re-evaluated for the appropriateness of additional therapy, and treatment is tailored to the individual patient based on goals, safety, perceived benefit, and performance status. Additional factors may also include how the patient responded to the initial therapy in terms of both the tolerance to treatment and time to recurrence. The National Comprehensive Cancer Network (NCCN) guidelines for central nervous system (CNS) tumors list acceptable second-line options for patients. These include pursuing a clinical trial, re-resection, additional systemic therapy, reirradiation with or without chemotherapy, and palliative/best supportive care among others (11). Even with this additional therapy, the median overall survival (OS) of patients with GB is less than 1 year (12). A second course of RT can be considered, especially in cases where there has been a long interval since the first course of RT was completed, the recurrence is reasonably sized, their performance status is good, a second course would be anticipated to be relatively safe, and the patient tolerated the first course well (13, 14).

As mentioned, RT has an important role in the treatment of newly diagnosed GB and potentially in the recurrent setting as well. With improving technology over the years, RT delivery has become more conformal and precise, allowing the field of radiation oncology to test shorter RT courses called hypofractionated RT (HFRT), usually defined as >2 Gy per fraction in fewer fractions compared to conventionally fractionated treatment. These courses are considered to be non-inferior for those 70 years and older or with a low performance status with a diagnosis of GB (15–17). These studies were the bases of the American Society for Radiation Oncology (ASTRO) guidelines that recommended HFRT for GB in patients greater than or equal to 70 years of age with fair-to-good performance status of greater than or equal to Karnofsky performance status (KPS) of 50 or in any patient with a poor KPS (18). The NCCN guidelines stratify treatment recommendations based on older or younger than 70 years and then by KPS (11). HFRT is not recommended in ages younger or equal to 70 years old with KPS greater than or equal to 60, but for all other categories, it is an option (11). The use of HFRT in younger patients with newly diagnosed GB is actively still under investigation. This includes the SAGA study, a randomized phase II trial comparing conventional and hypofractionated courses of RT in patients 18 and older (NCT05781321). In current practice, HFRT is usually offered to patients with older age and/or lower performance status.

Parallel to studies asking about HFRT for newly diagnosed GB, others have studied the risks and benefits of reirradiation for GB using hypofractionated approaches. This increasing body of evidence supports the idea that reirradiation of the brain in glioblastoma may be safe (13, 14, 19). It is important to note, however, that most reirradiation studies include patients who received initial treatment with 60 Gy in 30 fractions with concurrent TMZ, while some studies omitted the details associated with this initial RT regimen (13).

**Figure 1:**
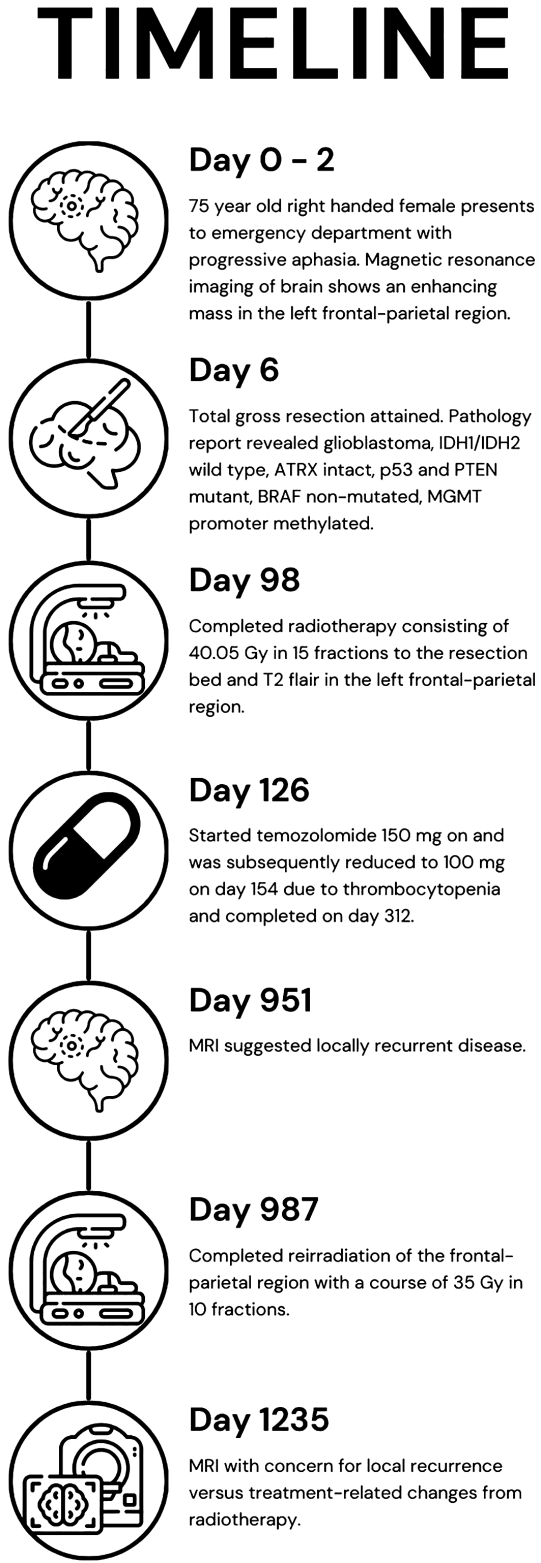
Timeline of the patient’s care from presentation (day 0), imaging (day 2), treatments (days 6, 98, and 126), recurrence (day 951), reirradiation (day 987), and question of recurrence or treatment-related changes (day 1,235).

To date, there is no standard treatment for patients that have recurrent GB. To our knowledge, this is the first case report to discuss the outcome of a patient who received HFRT for both first and second courses of RT. We believe that it is important to discuss a long-term survivor’s case to point out that factors beyond age and performance status could have important implications for select patients. Through this case report, we share a long-term survivor’s treatment timeline, ongoing at 42 months since diagnosis, who had two courses of HFRT as part of the management for GB (**Figure 1**).

## Clinical case

**Figure 2:**
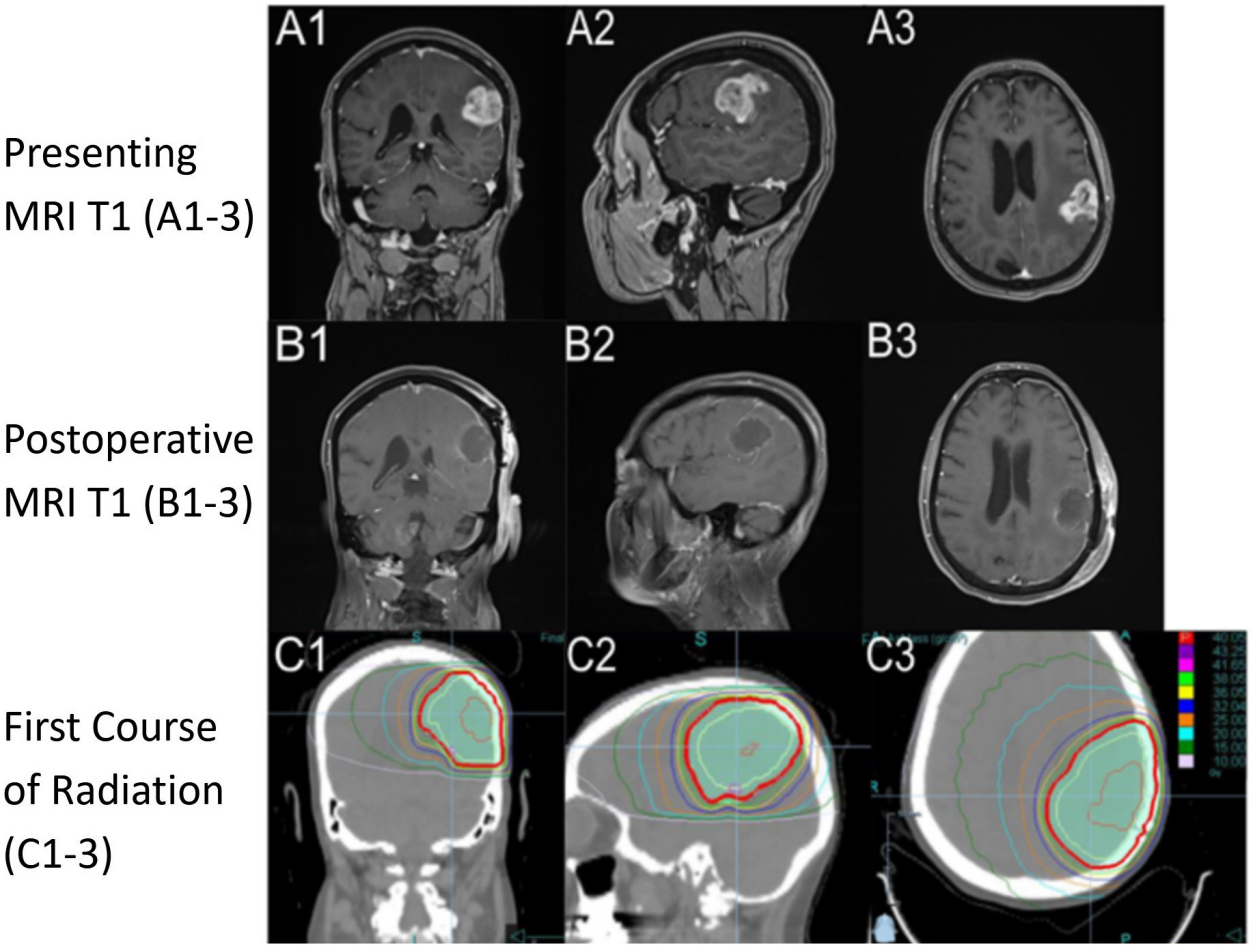
Imaging from presentation with T1 sequence on **(A1-A3)** demonstrating a hyperintense ring-enhancing lesion in the left frontal-parietal region. Postoperative imaging, day 7 after diagnosis, with T1 sequence on **(B1-B3)** demonstrating postoperative changes. RT plan 1 (RT1) utilizing a noncontrast computed tomography (CT) with dose color outline. First course of RT **(C1-C3)** (red is 40.05 Gy, purple is 43.25 Gy, pink 41.65 Gy, light green 38.05 Gy, yellow is 36.05 Gy, blue is 32.04 Gy, orange is 25.00 Gy, light blue is 20.00 Gy, dark green is 15.00 Gy, and light pink is 10.00 Gy). Column 1 is a coronal section through the body of caudate and anterior cerebellum, column 2 is a sagittal section through the left superficial temporal and parietal lobe, and column 3 is an axial section through superior cerebrum and lateral ventricles.

A 75-year-old, right-handed woman presented to the emergency department with progressive aphasia and a KPS of 90. She had no significant past medical history. She had a family history of treated skin carcinoma. Magnetic resonance imaging (MRI) of the brain T1 post-contrast revealed a 3.3 × 2.8 × 2.8-cm enhancing mass in the left frontal-parietal region and a second extra-axial contrast-enhancing lesion in the right occipital lobe with imaging features consistent with a meningioma (**Figures 2A1–3**). She underwent surgery on day 6 following her presentation where a total gross dissection was achieved. The pathology report confirmed WHO grade 4 glioblastoma, IDH1/IDH2 wild type, ATRX intact, p53 and PTEN mutant, BRAF non-mutated, and MGMT promoter methylated.

At 34 days after presentation, the neuro-oncologist saw the patient and recommended adjuvant chemoradiation with TMZ to tentatively start around day 60 following her presentation. However, her postoperative recovery was complicated by a deep vein thrombosis and pneumonia requiring hospitalization on day 37 where her performance status declined to a KPS of 60 and needed high-level respiratory support. She was treated at a tertiary center closer to her home and transferred on day 67 to our medical center. She went on to receive a course of HFRT of 4,005 cGy in 15 fractions (**Figures 2C1–3**) utilizing intensity-modulated radiotherapy (IMRT) without concurrent TMZ due to her tenuous clinical status. Her RT continued from day 75 to day 98. The patient’s performance status improved, and she was able to be transferred to a transitional care unit. Overall, she tolerated the treatment well, experiencing grade 1 fatigue from RT. On day 98, she had a seizure that required her to restart levetiracetam and start a 2-week course of dexamethasone. During and after her treatment, she was active in rehabilitation for deconditioning and aphasia. The patient expressed how her journey through recovery was very challenging but found joy in being able to read her books and interact with her family and friends as her condition improved.

**Figure 3:**
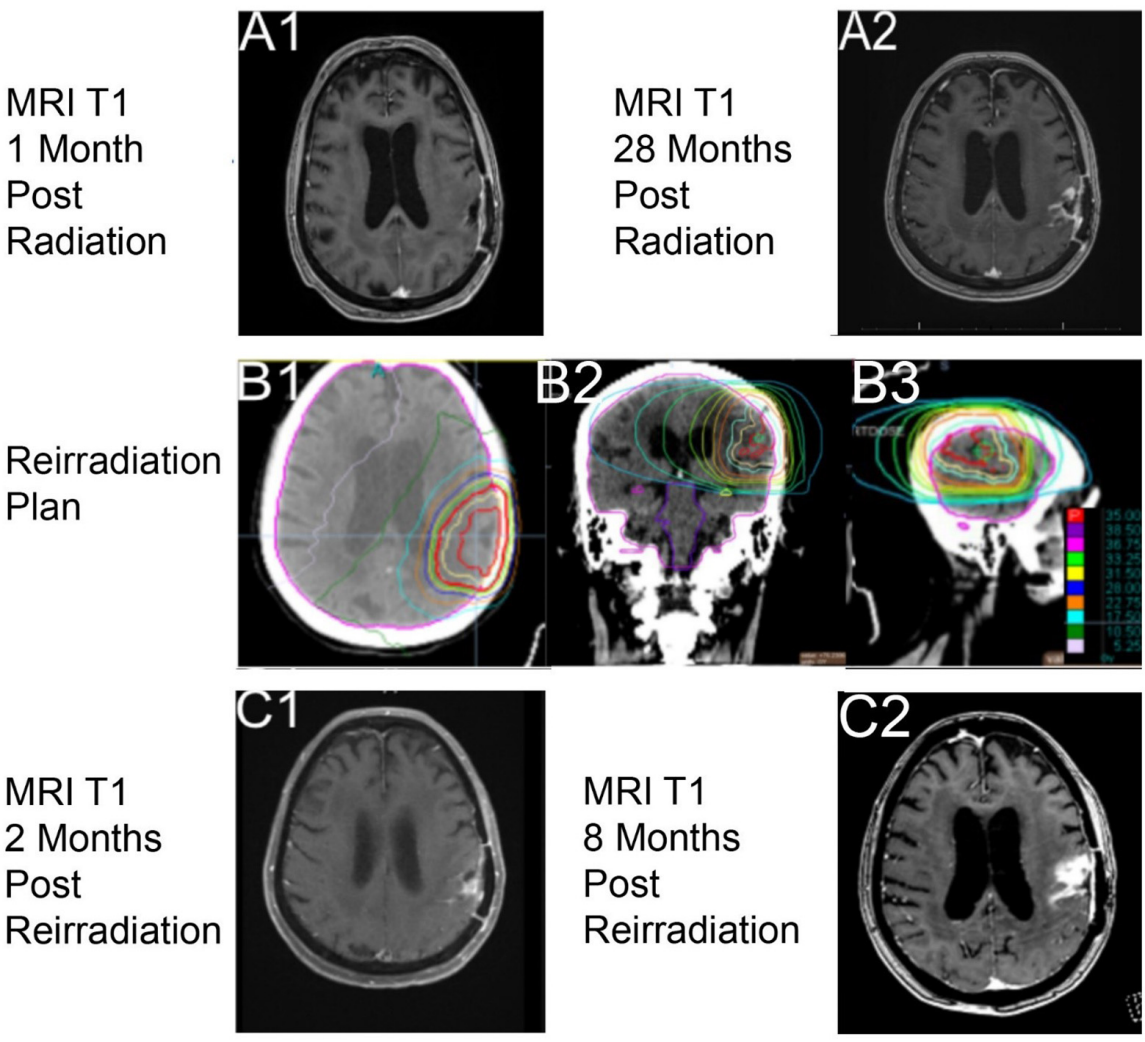
Imaging with T1 sequencing 1-month post-RT1, **(A1)** (axial through intraparietal sulcus), and 28 months post-radiation course 1, **(A2)** (axial through superior lateral ventricles). Reirradiation utilizing a noncontrast computed tomography (CT) with dose color outline and brain contoured as lavender, **(B1–B3)** (red is 35.00 Gy, purple is 38.50 Gy, pink 36.75 Gy, light green 33.25 Gy, yellow is 31.50 Gy, blue is 28.00 Gy, orange is 22.75 Gy, light blue is 17.50 Gy, dark green is 10.50 Gy, and light pink is 5.25 Gy). Imaging with T1 sequencing 2 months post-reirradiation, **(C1)** (axial through superior lateral ventricles). Imaging with T1 sequencing 8 months post-RT2, **(C2)** (axial through intraparietal sulcus).

A post-treatment MRI of the brain on day 125 was read by the diagnostic radiologist to have an interval increase in size of an enhancing parenchymal nodule just superior to the left parietal lobe resection cavity that was concerning for disease progression. The image findings at 4 weeks post-RT were discussed at a multidisciplinary tumor board and were felt to likely be treatment-related as her tumor was methylated (**Figure 3A1**). She started TMZ on day 126 at 150 mg/m^2^ that was reduced to 100 mg/m^2^ due to thrombocytopenia. TMZ was completed at about 6 months later, on day 312.

From the end of the patient’s RT, she was clinically improving and had stable 3-month interval imaging until an MRI on day 918 (27 months post-RT) showed a treatment-related change. At 1 month later, on day 951, MRI of the brain showed a marked progression in the parietal lobe within the previous radiation field (**Figure 3A2**). The multidisciplinary care team discussed all available options with the patient. Surgery was not recommended because of her age, a significant drop in performance status, and post-surgical complications after her first resection. Systemic therapy was not offered because of her poor performance status and the prior adverse hematologic effects from her first course of TMZ. RT was an option due to the size of recurrence, location of her disease, and favorable tolerance of the first course of RT. The patient was actively involved in shared decision-making and decided to proceed with a second course of RT. She desired to engage with a treatment but be able to interact with family and read her books for joy.

Reirradiation of 35 Gy in 10 fractions (**Figures 3B1–3**) was the selected fractionation for her second RT course. Unfortunately, she developed a seizure on day 960, before treatment, which led to a decrease in her KPS to 50. The patient was gradually improving, and she strongly wished to proceed with reirradiation, finishing on day 987. She tolerated this second course well, without acute toxicities, and maintained her post-seizure performance status of a KPS of 50.

The 2-month follow-up on day 1,025 with MRI of the brain (**Figures 3C1–2**) showed decreased multifocal nodular enhancement surrounding the margins of the left parietal resection cavity, persistent expansile T2 hyperintense signal surrounding the margins of the resection cavity, and expansile T2 hyperintense signal involving the left thalamus that appeared worsened compared to prior imaging. The follow-up imaging was stable until day 1,235 when there was a concern for local recurrent disease *versus* treatment-related changes from RT (**Figure 3C2**). At the time of this case report, the patient is living, but there is current concern for tumor recurrence on her most recent MRI, within the two prior RT fields.

## Discussion

This is a long-term survivor of GB who has survived 3.5 years and who underwent two HFRT courses with adjuvant TMZ at the time of new diagnosis. It is a rare occurrence for someone of this patient’s age to have survived for such a long time with this diagnosis; it is even more striking that she did so with a RT technique that has largely been reserved for those who do not survive very long after their tumor is identified. HFRT for newly diagnosed GB is mainly used for individuals who are older and/or have poor performance status. Because elderly patients unfortunately usually have a lower fitness, their expected shorter life expectancy after diagnosis and performance status at the time of recurrence can impact the perceived benefit from aggressive second-line treatment options, including a second course of RT. Unfortunately, it is not as likely that these patients will survive or be of the fitness needed to be offered a second course of RT.

There are certain prognostic factors that portend a longer survival that influenced our patient. She had a total gross resection, she received RT with adjuvant TMZ, and her GB has a methylated MGMT promoter. She also has factors that are associated with a poor prognosis, such as her older age (>70 years) and lower performance status, which are the reasons why her RT course was selected as such (20, 21). An important factor for her survival was most likely the methylated MGMT promoter status of her tumor that silences the creation of a DNA repair enzyme specifically for alkylation, such as the effects of TMZ. RTOG 0525 reported a mean OS of 21.2 months *versus* 14.0 months without methylation when treated with TMZ (22). TMZ works as an alkylating chemotherapy agent that damages DNA and has a synergistic effect along with RT. This combination results in a longer OS and PFS to patients with a methylated MGMT promoter (23). As such, concurrent and adjuvant TMZ is more beneficial compared to adjuvant alone (24). Our patient only received adjuvant TMZ due to her lower performance status at the time of RT, and the TMZ course started a month after her course finished. TMZ may have still been of benefit to the patient.

Risk factors beyond age and performance status are clearly important to predicting survival in patients with GB. As such, Zemskova et al. created a scoring system from a univariate analysis for factors that are associated with OS (25). They found that patients with a single lesion, maximum diameter <40 mm, KPS >90, MGMT promoter methylation, gross tumor resection, and addition of TMZ were significant in association to a 12-month OS and created a point system that separated patients into three groups: 32–35 points, 36–44 points, and 45–48 points. These three groups’ 12-month OS rates were 0%, 56%, and 92%, respectively, and suggested an ultra-hypofractionation (25 Gy in five fractions) for the first group, HFRT for the second, and conventional for the third, stating that each group would benefit the most from each of the different RT courses (25). Additional studies are needed to define other prognosis determinants for patients with GB. GB presents a significant challenge, as both the tumor and its treatments impact QOL. With no cure, the primary goal is to balance prolonging life while maintaining QOL. If this is the goal, then it would be beneficial for patients to have a shorter course to have more time doing what they enjoy and have a reduced financial burden (26). Studies have shown noninferiority using different hypofractionation regimens for elderly patients >70 years (16). Roa et al. used RT alone without TMZ, reporting a median OS of 5.6 months using 40.05 Gy in 15 fractions (16). Perry et al. compared HFRT (40.05 Gy in 15 fractions) with and without concurrent TMZ in 65- to 90-year-old patients that reported an OS of 9.3 and 7.6 months, respectively (5). These studies show that HFRT with concurrent TMZ can be of benefit in this age group.

Treatment for recurrent GB is complex and challenging as the benefits of treatment at this stage are usually less than a year. For this patient’s age, KPS, tolerance, and time from initial RT, reirradiation was selected as a favorable approach. A meta-analysis by Kazmi et al. (27) showed a pooled 6- and 12-month OS of 73% and 36% for patients receiving reirradiation, respectively (26). Along with the meta-analysis, the RTOG1205 study lead to a consensus that 35 Gy in 10 fractions is safe and effective for improving survival compared to systemic therapy alone (14). A secondary analysis of the RTOG trial 0525 stated a modest effect on OS compared to no therapy, but there was no significant survival difference between radiation compared to systemic therapy with or without radiation (22). Overall, this case demonstrates that HFRT may be a reasonable option for the upfront treatment of GB, as it did not preclude the patient from receiving a second HFRT course. Multiple factors, including tumor biology and genetics, may have played a role in this patient’s case and are the focus of current research. Prospective trials are needed to definitively identify the benefit of reirradiation after first-course HFRT in patients with GB.

## Data Availability

The datasets presented in this article are not readily available because of ethical and privacy restrictions. Requests to access the datasets should be directed to the corresponding author.

## References

[1] SEER*Explorer: An interactive website for SEER cancer statistics. Surveillance Research Program, National Cancer Institute (2024). Available at: https://seer.cancer.gov/statistics-network/explorer/.

[2] Stupp R Mason WP van den Bent MJ Weller M Fisher B Taphoorn MJ . Radiotherapy plus concomitant and adjuvant temozolomide for glioblastoma. New Engl J Med. (2005) 352:987–96. doi: 10.1056/NEJMoa043330 15758009

[3] Briceno N Vera E Komlodi-Pasztor E Abdullaev Z Choi A Grajkowska E . Long-term survivors of glioblastoma: Tumor molecular, clinical, and imaging findings. Neuro-oncology Adv. (2024) 6:vdae019. doi: 10.1093/noajnl/vdae019 PMC10901543 38420614

[4] Gulati S Jakola AS Nerland US Weber C Solheim O . The risk of getting worse: surgically acquired deficits, perioperative complications, and functional outcomes after primary resection of glioblastoma. World Neurosurg. (2011) 76:572–9. doi: 10.1016/j.wneu.2011.06.014 22251506

[5] Perry JR Laperriere N O’Callaghan CJ Brandes AA Menten J Phillips C . Short-course radiation plus temozolomide in elderly patients with glioblastoma. New Engl J Med. (2017) 376:1027–37. doi: 10.1056/NEJMoa1611977 28296618

[6] Lawrence YR Li XA el Naqa I Hahn CA Marks LB Merchant TE . Radiation dose-volume effects in the brain. Int J Radiat oncology biology Phys. (2010) 76:S20–7. doi: 10.1016/j.ijrobp.2009.02.091 PMC3554255 20171513

[7] Gibson D Ravi A Rodriguez E Chang S Oberheim Bush N Taylor J . Quantitative analysis of MGMT promoter methylation in glioblastoma suggests nonlinear prognostic effect. Neuro-oncology Adv. (2023) 5:vdad115. doi: 10.1093/noajnl/vdad115 PMC10611422 37899778

[8] Ening G Osterheld F Capper D Schmieder K Brenke C . Risk factors for glioblastoma therapy associated complications. Clin Neurol Neurosurg. (2015) 134:55–9. doi: 10.1016/j.clineuro.2015.01.006 25942630

[9] Wick W Platten M Meisner C Felsberg J Tabatabai G Simon M . Temozolomide chemotherapy alone versus radiotherapy alone for Malignant astrocytoma in the elderly: the NOA-08 randomised, phase 3 trial. Lancet Oncol. (2012) 13:707–15. doi: 10.1016/S1470-2045(12)70164-X 22578793

[10] Caruso R Pesce A Wierzbicki V . A very rare case report of long-term survival: A patient operated on in 1994 of glioblastoma multiforme and currently in perfect health. Int J Surg Case Rep. (2017) 33:41–3. doi: 10.1016/j.ijscr.2017.02.025 PMC5338899 28273605

[11] Nabors LB Portnow J Baehring J Bhatia A Bloch O Brem S . Central nervous system cancers version: 2.2024. National Comprehensive Cancer Network (NCCN) (2024). NCCN clinical practice guidelines in oncology. 10.6004/jnccn.2020.0052 33152694

[12] Vaz-Salgado MA Villamayor M Albarrán V Alía V Sotoca P Chamorro J . Recurrent glioblastoma: A review of the treatment options. Cancers. (2023) 15:4279. doi: 10.3390/cancers15174279 37686553 PMC10487236

[13] Shen CJ Kummerlowe MN Redmond KJ Martinez-Gutierrez JC Usama SM Holdhoff M . Re-irradiation for Malignant glioma: Toward patient selection and defining treatment parameters for salvage. Adv Radiat Oncol. (2018) 3:582–90. doi: 10.1016/j.adro.2018.06.005 PMC6200913 30370358

[14] Tsien CI Pugh SL Dicker AP Raizer JJ Matuszak MM Lallana EC . NRG oncology/RTOG1205: A randomized phase II trial of concurrent bevacizumab and reirradiation versus bevacizumab alone as treatment for recurrent glioblastoma. J Clin oncology: Off J Am Soc Clin Oncol. (2023) 41:1285–95. doi: 10.1200/JCO.22.00164 PMC9940937 36260832

[15] Malmström A Grønberg BH Marosi C Stupp R Frappaz D Schultz H . Temozolomide versus standard 6-week radiotherapy versus hypofractionated radiotherapy in patients older than 60 years with glioblastoma: the Nordic randomised, phase 3 trial. Lancet Oncol. (2012) 13:916–26. doi: 10.1016/S1470-2045(12)70265-6 22877848

[16] Roa W Brasher PM Bauman G Anthes M Bruera E Chan A . Abbreviated course of radiation therapy in older patients with glioblastoma multiforme: a prospective randomized clinical trial. J Clin oncology: Off J Am Soc Clin Oncol. (2004) 22:1583–8. doi: 10.1200/JCO.2004.06.082 15051755

[17] Roa W Kepka L Kumar N Sinaika V Matiello J Lomidze D . International atomic energy agency randomized phase III study of radiation therapy in elderly and/or frail patients with newly diagnosed glioblastoma multiforme. J Clin oncology: Off J Am Soc Clin Oncol. (2015) 33:4145–50. doi: 10.1200/JCO.2015.62.6606 26392096

[18] Cabrera AR Kirkpatrick JP Fiveash JB Shih HA Koay EJ Lutz S . Radiation therapy for glioblastoma: Executive summary of an American Society for Radiation Oncology Evidence-Based Clinical Practice Guideline. Pract Radiat Oncol. (2016) 6:217–25. doi: 10.1016/j.prro.2016.03.007 27211230

[19] Combs SE Thilmann C Edler L Debus J Schulz-Ertner D . Efficacy of fractionated stereotactic reirradiation in recurrent gliomas: long-term results in 172 patients treated in a single institution. J Clin oncology: Off J Am Soc Clin Oncol. (2005) 23:8863–9. 10.1200/JCO.2005.03.4157 16314646

[20] Brown NF Ottaviani D Tazare J Gregson J Kitchen N Brandner S . Survival outcomes and prognostic factors in glioblastoma. Cancers. (2022) 14:3161. doi: 10.3390/cancers14133161 35804940 PMC9265012

[21] Melhem JM Detsky J Lim-Fat MJ Perry JR . Updates in IDH-wildtype glioblastoma. Neurotherapeutics: J Am Soc Exp Neurother. (2022) 19:1705–23. doi: 10.1007/s13311-022-01251-6 PMC9154038 35641844

[22] Shi W Scannell Bryan M Gilbert MR Mehta MP Blumenthal DT Brown PD . Investigating the effect of reirradiation or systemic therapy in patients with glioblastoma after tumor progression: A secondary analysis of NRG oncology/radiation therapy oncology group trial 0525. Int J Radiat oncology biology Phys. (2018) 100:38–44. doi: 10.1016/j.ijrobp.2017.08.038 PMC5742545 29102648

[23] Minniti G Salvati M Arcella A Buttarelli F D’Elia A Lanzetta G . Correlation between O6-methylguanine-DNA methyltransferase and survival in elderly patients with glioblastoma treated with radiotherapy plus concomitant and adjuvant temozolomide. J neuro-oncology. (2011) 102:311–6. doi: 10.1007/s11060-010-0324-4 20686820

[24] Sher DJ Henson JW Avutu B Hochberg FH Batchelor TT Martuza RL . The added value of concurrently administered temozolomide versus adjuvant temozolomide alone in newly diagnosed glioblastoma. J neuro-oncology. (2008) 88:43–50. doi: 10.1007/s11060-008-9530-8 PMC2658810 18231723

[25] Zemskova O Yu NY Trillenberg P Bonsanto MM Leppert J Rades D . Identification of patients with glioblastoma who may benefit from hypofractionated radiotherapy. Anticancer Res. (2023) 43:2725–32. doi: 10.21873/anticanres.16439 37247904

[26] Ghosh S Baker S de Castro DG Kepka L Kumar N Sinaika V . Improved cost-effectiveness of short-course radiotherapy in elderly and/or frail patients with glioblastoma. Radiotherapy oncology: J Eur Soc Ther Radiol Oncol. (2018) 127:114–20. doi: 10.1016/j.radonc.2018.01.017 29452901

[27] Kazmi F Soon YY Leong YH Koh WY Vellayappan B . Re-irradiation for recurrent glioblastoma (GBM): a systematic review and meta-analysis. J neuro-oncology. (2019) 142:79–90. doi: 10.1007/s11060-018-03064-0 30523605

